# Important Advice

**Published:** 1878-05

**Authors:** 


The great curative principle, to which the reader's attention is specially solicited, is this: In any attack of consumption, or its repetition, the patient should hope it was the last cluster to soften, and that if he can only weather this storm, it may be the last, and life and happiness may be his, for long years to come. Too much attention can scarcely be paid to this idea, and we hope every invalid reader will sleep on it nightly, and make it the ground of active, strenuous effort for health, every succeeding day, even until life's close, for the truly brave die striving. This being their motto, they do, in these diseases often, very often, outlive the prognostications of ignorance and presumption, for it is only such who can peril a prophecy of recovery or death; they speak firmly, where the physician gives opinions with trembling on the tongue.

Another practical fact, it is difficult to refrain from mentioning here:

On the partial or complete recovery from an attack of consumption, do not, as you value life, intermit a single possible effort for maintaining the highest possible degree of health; keep it up, until a habit of health is established, and even then, until the close of life, make it your study to live rationally, apportioning your eating to your exercise, as true wisdom dictates.

The symptoms of consumption have been described in a general manner. It is purposed under this head to speak of the far-off symptoms, which, if promptly treated, may eventuate in cure, with as much certainty as belongs to ordinary diseases. It is scarcely to be hoped that any attention will be paid to these, yet a book on this subject could not have claim to completeness of history without discoursing something on this head. It is certainly desirable, for it is highly practical, capable as it is, of making Consumption of the Lungs a manageable disease. But it is sadly feared, that it is to be the consummation of future centuries.

It is with consumption as it is with cholera, easily manageable in its first stages; in its last, utterly incurable. All men looked with horror on the Asiatic curse when it first visited our shores. When it was first described as sweeping the world with death, it was represented to be as instantaneous as a plague or palsy, and without one single warning note, hurrying multitudes to the grave; and yet on more minute and scientific inquiry, it is an established fact, that cholera, when attended to, in its premonitory stages, is an easily manageable disease, and is now shorn of half its horrors.

So of consumption, if we took note of its far off symptoms, and would then enter upon a course of life wisely energetic, it becomes one of the most manageable of diseases. The important practical inquiry then arises, what are the earliest and most invariable symptoms of common consumption of the lungs?

*Cough* is not an early symptom of consumption, necessarily, for there are many cases on record, in which cough was not an observed symptom, until within two or three weeks of death, and on examination, the lungs presented a diseased mass, burrowed with cavities.

*Spitting blood* is not an early symptom of consumption, necessarily, for about one-third of those who die of that disease, do not spit blood at all.

Among the very earliest symptoms of forming consumption, are combinations of the following; not all, perhaps, observable in any one case: A quicker pulse than common, a paler face, easily chilled after eating, more readily put out of breath than common, less fullness of flesh than usual at the corresponding season of the year, an unusual feeling of unrest on getting up in the morning, a greater tendency to coldness of the hands and feet, every now and then a day passing without any action of the bowels, with a very bad taste in the mouth when first waking up in the morning; a cold is easily taken, is more frequent, and lasts longer and longer, until one cold runs into another, making the confirmed cough, so ominous of approaching ill.

It will be seen at a single glance, from these symptoms, that they all indicate one thing, and that one thing is at the bottom of every case of consumption—*a want of vitality;* that is, a want of general vigor of system, of constitution.

But of all the things named, it will be more practical to select the two which are seldom, if ever absent, in any of the above combinations which result in consumption; hence it is import ant to be at some pains in stating them in their bearings. A quick pulse and a short breath pervade the disease from its earliest beginnings, during its entire progress, and down to its fatal end. Multitudes of lives might be saved yearly, if these two symptoms were promptly and wisely attended to. The importance of so doing, no language can adequately portray, and if it did, the people would not attend to it, with only here and there an exception. But a great truth is of small seed and of slow growth; yet that growth is certain, and its spread uncontrollable—the more so, as education becomes more general.

